# The Edmonton Symptom Assessment System is a valid, reliable, and responsive tool to assess symptom burden in decompensated cirrhosis

**DOI:** 10.1097/HC9.0000000000000385

**Published:** 2024-03-18

**Authors:** John Donlan, Chengbo Zeng, Teresa Indriolo, Lucinda Li, Enya Zhu, Joyce Zhou, Kedie Pintro, Nora Horick, Maria Edelen, Raymond T. Chung, Areej El-Jawahri, Nneka N. Ufere

**Affiliations:** 1 Harvard Medical School, Boston, Massachusetts, USA; 2 Patient-Reported Outcomes, Value, and Experience (PROVE) Center, Brigham and Women’s Hospital, Boston, Massachusetts, USA; 3 Department of Medicine, Liver Center, Gastrointestinal Division, Massachusetts General Hospital, Boston, Massachusetts, USA; 4 Department of Internal Medicine, Massachusetts General Hospital, Boston, Massachusetts, USA; 5 MGH Biostatistics, Massachusetts General Hospital, Boston, Massachusetts, USA; 6 Department of Medicine, Division of Hematology and Oncology, Massachusetts General Hospital, Boston, Massachusetts, USA

## Abstract

**Background:**

While there is a growing need for interventions addressing symptom burden in patients with decompensated cirrhosis (DC), the lack of validated symptom assessment tools is a critical barrier. We investigated the psychometric properties of the revised Edmonton Symptom Assessment System (ESAS-r) in a longitudinal cohort of patients with DC.

**Methods:**

Adult outpatients with DC were prospectively recruited from a liver transplant center and completed ESAS-r at baseline and week 12. We examined reliability, floor/ceiling effects, structural validity, and known-groups validity. We examined the convergent and predictive validity of ESAS-r with health-related quality of life using the Short Form Liver Disease Quality of Life (SF-LDQOL) and responsiveness to changes in anxiety and depression using the Hospital Anxiety and Depression Scale and Patient Health Questionnaire-9 from baseline to week 12.

**Results:**

From August 2018 to September 2022, 218 patients (9% Child-Pugh A, 59% Child-Pugh B, and 32% Child-Pugh C) were prospectively recruited and completed the ESAS-r, SF-LDQOL, Patient Health Questionnaire-9, and Hospital Anxiety and Depression Scale at baseline and week 12 (n = 135). ESAS-r had strong reliability (Cronbach’s alpha 0.86), structural validity (comparative fit index 0.95), known-groups validity (Child-Pugh A: 25.1 vs. B: 37.5 vs. C: 41.4, *p* = 0.006), and convergent validity (r = −0.67 with SF-LDQOL). Floor effects were 9% and ceiling effects were 0.5%. Changes in ESAS-r scores from baseline to week 12 significantly predicted changes in SF-LDQOL (β = −0.36, *p* < 0.001), accounting for 30% of the variation. ESAS-r was strongly responsive to clinically meaningful changes in SF-LDQOL, Patient Health Questionnaire-9, and Hospital Anxiety and Depression Scale.

**Conclusions:**

ESAS-r is a reliable, valid, and responsive tool for assessing symptom burden in patients with DC and can predict changes in health-related quality of life. Future directions include its implementation as a key outcome measure in cirrhosis care and clinical trials.

## Graphical abstract

**Figure:**
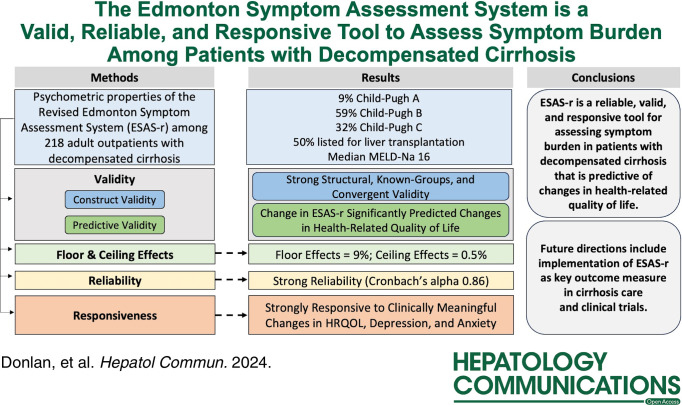

## INTRODUCTION

Patients with decompensated cirrhosis (DC) experience poor health-related quality of life (HRQOL) not only due to complications of liver disease such as hepatic encephalopathy (HE), ascites, and variceal hemorrhage but also due to high physical and psychological symptom burden.^1^ Symptoms such as pain, fatigue, sleep disturbances, nausea, depression, and anxiety are both highly prevalent and undertreated.^1^,^2^ An important developing priority in cirrhosis care is the need for interventions to reduce physical and psychological symptom burden, which has the potential to significantly improve HRQOL for patients with DC.^3^,^4^ However, a key barrier to achieving this goal is the lack of a simple and clinically relevant tool for assessing a diverse set of symptoms that is reliable, responsive to change, and validated among patients with DC.^3^,^5^,^6^ Additionally, the relative contribution of symptom burden to HRQOL among patients with DC is not yet well understood.

The revised Edmonton Symptom Assessment System (ESAS-r) is a simple 10-item measure that uses a numerical rating scale to assess physical and psychological symptom severity in patients with serious illnesses.^7^ Originally developed for inpatient palliative care, the ESAS-r has since been validated in numerous populations, including patients with advanced cancers and end-stage renal disease.^7^,^8^ However, no prior study has investigated the psychometric properties of the ESAS-r among patients with DC. In this prospective longitudinal cohort study of 218 ambulatory patients with DC, we sought to assess: (1) the reliability, floor and ceiling effects, and construct validity of the ESAS-r; (2) the predictive validity of ESAS-r for HRQOL as measured by the Short Form Liver Disease Quality of Life (SF-LDQOL); and (3) the responsiveness of ESAS-r to clinically meaningful changes in HRQOL, and depression and anxiety in this population.

## METHODS

### Study population

From August 2018 to September 2022, patients were consecutively recruited from Massachusetts General Hospital during routine outpatient hepatology clinical visits. Eligibility criteria included: (1) age ≥18 y old; (2) a diagnosis of cirrhosis complicated by ascites, HE, and/or gastro esophageal variceal bleed; and (3) the ability to read questions in English. Exclusion criteria included: (1) history of prior liver transplantation; (2) presence of uncontrolled hepatic encephalopathy (> West Haven stage 1), psychiatric disorder, dementia, or other cognitive impairment precluding ability to provide informed consent as determined by their primary hepatologist; (3) presence of HCC beyond Milan criteria^9^; (4) current history of extrahepatic malignancy (excluding nonmelanoma skin cancer); and (5) currently receiving palliative care or hospice care as these patients were more likely to be receiving symptom-focused care. Study staff communicated directly with clinicians caring for potential study participants daily to confirm patients who were eligible and exclude those who were not prior to the approach. Additionally, patients were excluded if study staff deemed them unable to provide informed consent.

Self-reported gender, race, ethnicity, relationship status, educational attainment, household income, living situation, and employment status were collected at enrollment. Clinical characteristics including etiology of liver disease (alcohol; metabolic dysfunction–associated steatotic liver disease; viral hepatitis B or C; or other), cirrhosis complications (ascites, HE, esophageal variceal bleed), and severity (Model for End-Stage Liver Disease-Sodium [MELD-Na]score; and Child-Pugh score that were available closest to the time of completion of baseline survey), transplant listing status at enrollment (listed vs. not listed), and clinical comorbidities as measured by a cirrhosis-specific comorbidity (CirCom) scoring system^10^ were extracted from the electronic medical record at the time of enrollment through chart review by the research team. All research was conducted in accordance with both the Declarations of Helsinki and the Istanbul Study. Study participants did not receive remuneration for study participation. All study participants provided written informed consent and the Mass General Brigham Institutional Review Board approved this study.

### Patient-reported outcome measures

Eligible patients had up to 30 days after providing informed consent to complete baseline study questionnaires either verbally in person or by telephone, on paper, or electronically. Patients who consented were considered enrolled upon completion of baseline study questionnaires, which included the completion of patient-reported outcome measures (PROMs) as described in more detail below. Enrolled patients were contacted at week 12 after their study enrollment to complete the same set of 4 PROMs. The full survey battery is available in Supplemental Material, http://links.lww.com/HC9/A796.

#### Edmonton Symptom Assessment System revised (ESAS-r)

We assessed patient-reported symptom burden using ESAS-r. The ESAS-r measures the severity of nine common symptoms that patients with serious illnesses experience (pain, tiredness, drowsiness, nausea, lack of appetite, shortness of breath, depression, anxiety, and sensation of well-being) with the option of adding a tenth patient-specific symptom. Content validity for the ESAS-r in patients with DC was assessed by expert, patient, and literature review, and we subsequently included muscle cramps as the tenth item as it is a highly prevalent symptom among patients with cirrhosis, which is associated with poor HRQOL.^11^,^12^ Patients reported their symptom severity over the past 7 days using a numerical rating scale ranging from 0 to 10 (0 reflecting absence of the symptom; 10, the worst possible severity) for each item. We calculated a total symptom distress score (range 0–100, with higher scores indicating higher symptom burden) by summing the 10 individual item scores as described.^7^ The minimal clinically important difference (MCID) for ESAS-r total score is 3–4 points.^13^

#### Short Form Liver Disease Quality of Life (SF-LDQOL) questionnaire

We assessed patients’ disease-specific HRQOL using the SF-LDQOL questionnaire. The SF-LDQOL questionnaire combines the Short Form-36 with the Liver Disease Quality of Life instrument and has been validated in patients with DC.^14^,^15^ It is able to detect clinically meaningful changes in QOL over time and predict survival in patients with DC.^16^ The SF-LDQOL includes a total of 14 questions (36 items) measuring 9 domains including symptoms of liver disease, effects of liver disease, concentration/memory, health distress, sleep, loneliness, hopelessness, stigma of liver disease, and sexual functioning/problem. Each item in the SF-LDQOL is scored on a Likert scale with a higher total sum score reflecting higher HRQOL. Across the 9 domains, items are normalized to a scale of 0–100 before calculating the mean score for each domain. We derived the total score of SF-LDQOL (range 0–100, with higher scores indicating better HRQOL) by averaging the mean scores from the nine domains as described.^15^

#### Hospital Anxiety and Depression Scale (HADS)

We assessed patients’ self-reported anxiety and depression using the 14-item Hospital Anxiety and Depression Scale (HADS).^17^ The HADS consists of two 7-item subscales assessing symptoms of anxiety (HADS-Anxiety) and depression (HADS-Depression) using a 4-option Likert response scale, with subscale scores ranging from 0 (no distress) to 21 (maximum distress).

#### Patient Health Questionnaire-9 (PHQ-9)

We used the 9-item PHQ-9 (range 0–27) to detect symptoms of major depressive disorder in the past 2 weeks according to the criteria of the Diagnostic and Statistical Manual of Mental Disorders (Fourth Edition), with higher scores indicating worse depression.^18^

### Statistical analysis

We describe the continuous demographic and clinical variables using median and IQR and categorical variables using frequencies and percentages. For each PROM, we report the mean and standard deviation at baseline. For each item of the ESAS-r scale, we report the percentage of patients with moderate-severe symptom burden, which is defined as an individual item score ≥ 4.^7^ Factor analysis was conducted using Mplus version 8.7 (Muthén & Muthén, Los Angeles, CA), while all other analyses were performed using SAS version 9.4 (SAS Institute, Inc., Cary, NC). Statistical significance was determined by a two-sided p-value of less than 0.05 or a 95% confidence interval that did not include 1.

#### Reliability

We evaluated the internal consistency of ESAS-r by calculating Cronbach’s alpha (value ≥ 0.70 indicates good internal consistency) using the 10 items at baseline and at week 12.^19^

#### Floor and ceiling effects

We evaluated the floor and ceiling effects for the ESAS-r total score at baseline by identifying the percentage of patients that either had the lowest or the highest possible scores. We used the commonly accepted threshold of 15% of participants achieving total ESAS-r scores between 0 and 10 (floor effect) or between 90 and 100 (ceiling effect).^20^

#### Construct validity

We evaluated the construct validity of ESAS-r based on (1) structural validity, (2) convergent validity, (3) divergent validity, and (4) known-groups validity.

We used confirmatory factor analysis to evaluate whether the items reflected the concept of symptom burden in patients with DC (structural validity). We evaluated structural validity using the chi-square value and used the root mean square error of approximation (cutoff value < 0.06), comparative fit index (cutoff value > 0.95), Tucker-Lewis index (cutoff value > 0.95), and standardized root mean square residual (cutoff value < 0.08) to identify good model fit.^21^ As the chi-square value is sensitive to sample size, we used the ratio of chi-square to its degree of freedom to evaluate the model fit; a ratio of ≤ 3 indicates a good model fit.^22^ We modified the factor model based on the modification indices and empirical evidence regarding the correlations between individual symptoms (eg, tiredness and drowsiness, depression and anxiety).^22^,^23^

To evaluate convergent and divergent validities, we calculated the product-moment correlations of the ESAS-r total score with other PROMs (SF-LDQOL, HADS-Anxiety, HADS-Depression, and PHQ-9 for convergent validity) and with MELD-Na scores (divergent validity) for all patients at baseline. We additionally calculated the correlations of depression on ESAS-r with HADS-Depression and PHQ-9, and anxiety on ESAS-r with HAD-Anxiety. Correlation coefficients were classified as follows: weak (< 0.4), moderate (0.4–0.7), and strong (> 0.7).^24^

We evaluated the known-groups validity using independent sample t-tests (presence of HE vs. no, presence of ascites vs. no) or ANOVA (Child-Pugh A vs. B vs. C) to identify group differences by ESAS-r total score.

#### Responsiveness

We evaluated the external responsiveness of ESAS-r by calculating the mean differences and 95% CIs in total scores between baseline and week 12 for patients with or without clinically meaningful improvement or worsening on PHQ-9, HADS-Depression, HADS-Anxiety, and SF-LDQOL scores over the same time period using complete case analysis.^25^ The MCID is 5 points for PHQ-9 and 1.5 points for HADS-Depression and HADS-Anxiety.^26^,^27^ For SF-LDQOL, we used an MCID cutoff of 8 based on an ongoing clinical trial on the treatment of ascites using SF-LDQOL as the primary outcome (REDUCe 2 Study).^6^

#### Predictive validity

**FIGURE 1:**
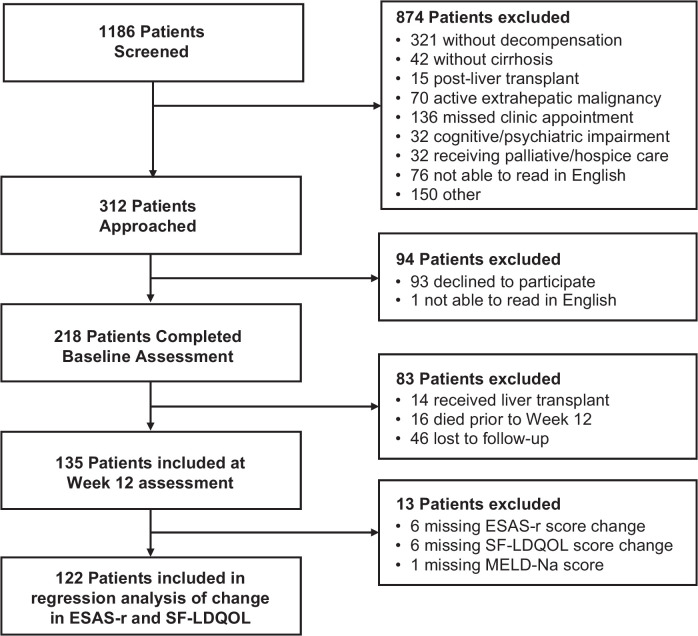
Patient Flowchart. Abbreviation: ESAS-r, Edmonton Symptom Assessment System; MELD-Na, Model for End-Stage Liver Disease-Sodium; SF-LDQOL, Short Form Liver Disease Quality of Life.

We examined whether the change of symptom burden measured by ESAS-r predicted the change of HRQOL measured by SF-LDQOL between baseline and week 12 first using univariate regression analysis among patients who provided complete case data at both timepoints (n = 122 in Figure 1). We calculated the *R* ^2^ to evaluate how much of the variation of change of SF-LDQOL was explained by the change of symptom burden as measured by the ESAS-r total score. We ran a multivariable analysis to examine this relationship after adjusting for the following potential confounders determined a priori or on a review of empirical evidence given their associations with HRQOL: age; MELD-Na score; presence of ascites or HE; diagnosis of alcohol; transplant listing status; presence of HCC; and CirCom comorbidity score.^28^

#### Sensitivity analyses

For patients who did not fill the sexual functioning/problem domain of the SF-LDQOL at baseline or week 12 follow-up (n = 14), their SF-LDQOL total scores were computed based on the remaining 8 domains. We ran a sensitivity analysis in which sexual functioning was excluded from the calculation of the total score of SF-LDQOL in all study patients. We replicated the regression analyses above for predictive validity using the change of SF-LDQOL that was calculated without sexual functioning.

Across all analyses, as the amount of missing data was small (< 10%), we used complete case analysis to handle missingness.

## RESULTS

### Overview

A total of 1186 participants were screened for eligibility. We approached 312 participants and enrolled 218 participants (69.9%) (Figure 1). At baseline, 98.6% (n = 215) of the participants self-completed the surveys and 1.4% (n = 3) had the surveys administered to them verbally. Participants had a median age of 60 years (IQR: 51–65 y) and the majority were White (89.0%, n = 194). Half of the patients were actively listed for liver transplantation (50%, n = 109) with a median MELD-Na of 16 and the following distribution of Child-Pugh classes: A (8.7%, n = 19), B (59.2%, n = 129), and C (32.1%, n = 70). There were 135 patients at week 12 follow-up: 83 patients who died, underwent liver transplant, or were lost to follow-up were excluded from the analysis in week 12. The 83 patients who were excluded from the week 12 analysis had significantly higher MELD-Na (Median: 18 [IQR: 13–25] vs. 15 [IQR: 11–19], *p* = 0.001) and Child-Pugh (Median: 9 [IQR: 8–11] vs. 8 [IQR: 7–10], *p* = 0.015) scores at baseline. All patients at week 12 self-completed the surveys. Other sociodemographic and clinical characteristics are shown in Table 1.

**TABLE 1:** Demographic and clinical characteristics of the analytic samples

Demographic and clinical characteristics	Baseline (N = 218)	Week 12 (N = 135)
Age (Median, IQR, range)	60 (51–65; 27–74)	59 (51–65; 27–74)
Male	118 (54.1)	79 (58.5)
Race		
White	194 (89.0)	121 (89.6)
African American or Black	5 (2.3)	3 (2.2)
Other	18 (8.3)	10 (7.5)
Missing	1 (0.4)	1 (0.7)
Hispanic or Latino	6 (2.8)	2 (1.5)
Married or living with someone as if married	139 (63.8)	88 (65.2)
Educational level		
High school graduate or lower	71 (32.6)	38 (28.2)
Some college	61 (28.0)	37 (27.4)
College graduate or higher	86 (39.4)	60 (44.4)
Annual household income		
$50,000 or less	103 (47.3)	63 (46.7)
More than $50,000	107 (49.1)	68 (50.3)
Missing	8 (3.6)	4 (3.0)
Liver disease etiology		
Alcohol	127 (58.3)	77 (57.0)
MASLD	50 (22.9)	32 (23.7)
HBV or HCV	29 (13.3)	17 (12.6)
Other	12 (5.5)	9 (6.7)
MELD-Na score (median, IQR, range)	16 (11–22; 6–40)	15 (11–19; 6–32)
Decompensation		
Ascites	200 (91.7)	122 (90.4)
HE	161 (73.9)	101 (74.8)
Esophageal variceal bleed	72 (33.0)	43 (31.9)
Child-Pugh score (median, IQR, range)	9 (7–10; 5–14)	8.0 (7–10; 5–13)
A (5–6)	19 (8.7)	11 (8.2)
B (7–9)	129 (59.2)	89 (65.9)
C (10–15)	70 (32.1)	35 (25.9)
Transplant status		
Actively listed for transplant	109 (50.0)	76 (56.3)
Evaluated but not listed	6 (2.3)	2 (1.5)
No evaluation	103 (47.7)	57 (42.2)
HCC at enrollment		
No	193 (88.5)	122 (90.4)
Yes	25 (11.5)	13 (9.6)

Abbreviations: HE, hepatic encephalopathy; HCC, hepatocellular carcinoma; MASLD, metabolic dysfunction–associated steatotic liver disease; MELD-Na, Model for End-Stage Liver Disease-Sodium.

**FIGURE 2:**
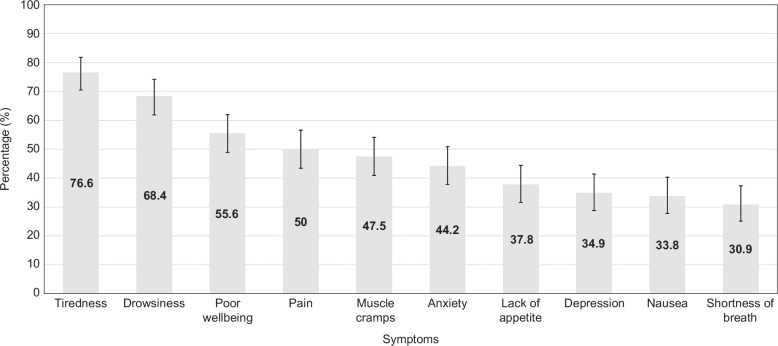
Percentage of patients with moderate-to-severe symptoms on ESAS-r at baseline. Abbreviation: ESAS-r, Edmonton Symptom Assessment System.

Mean (standard deviation) scores for PROMs at baseline are shown in Table 2. At baseline, complete cases were available for the PROMs as follows: ESAS-r (n = 213), SF-LDQOL (n = 209), HADS-Depression (n = 210), HADS-Anxiety (n = 213), PHQ-9 (n = 205). At baseline, the percentage of patients reporting moderate-to-severe symptoms on ESAS-r was highest for tiredness (76.6%, n = 167), drowsiness (68.4%, n = 149), poor well-being (55.6%, n = 120), pain (50%, n = 109), and muscle cramps (47.5%, n = 103). The percentages of patients reporting moderate-to-severe symptoms for each ESAS-r item are shown in Figure 2 and Supplemental Table E1, http://links.lww.com/HC9/A797.

**TABLE 2:** Mean scores on patient-reported outcome measures at baseline

Patient-reported outcomes	Mean (SD)
ESAS-r	37.7 (19.9)
SF-LDQOL	58.6 (16.4)
HADS-Depression	6.7 (4.1)
HADS-Anxiety	7.2 (4.3)
PHQ-9	8.6 (5.8)

Abbreviations: ESAS-r, revised Edmonton Symptom Assessment System; HADS, Hospital Anxiety and Depression Scale; PHQ-9, Patient Health Questionnaire-9; SF-LDQOL, Short Form Liver Disease Quality of Life.

### Reliability

The ESAS scale had adequate internal consistency at baseline (Cronbach’s alpha = 0.86) and week 12 (Cronbach’s alpha = 0.90).

### Floor and ceiling effects

At baseline, floor effects were 8.5% (n = 18) and ceiling effects were 0.5% (n = 1) for the ESAS-r total score. Among the 133 patients who completed the week 12 assessment, floor effects were 9% (n = 12) and ceiling effects were 0.8% (n = 1). Among the 83 patients who either died, underwent liver transplantation, or were lost to follow-up at week 12, a total of 80 provided complete cases for ESAS-r at baseline. Within this group, floor effects were 7.5% (n = 6) and ceiling effects were 0% (n = 0).

### Construct validity

**FIGURE 3:**
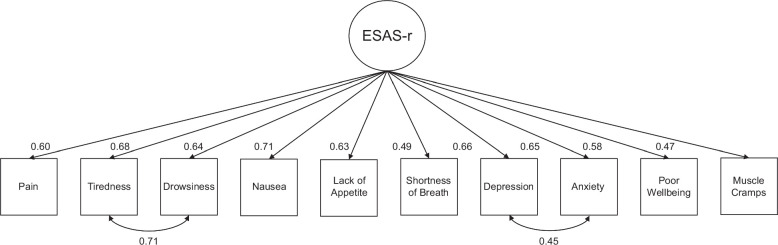
Factor structure of the ESAS-r. Abbreviation: ESAS-r, Edmonton Symptom Assessment System.

Structural validity. The confirmatory factor analysis for the ESAS-r scale showed an acceptable model fit after adding the correlations between depression and anxiety and drowsiness and tiredness (chi-square/df = 2.4, comparative fit index = 0.95, Tucker-Lewis index = 0.93, root mean square error of approximation = 0.08, standardized root mean square residual = 0.05). The factor loadings ranged from 0.47 in muscle cramps to 0.71 in nausea. Figure 3 shows the factor structure and loadings for the ESAS-r scale.

Convergent and divergent validity. ESAS-r had moderate-to-strong correlations with SF-LDQOL (r = −0.67), HADS-Depression (r = 0.61), HADS-Anxiety (r = 0.63), and PHQ-9 (r = 0.72) but was not correlated with MELD-Na score (r = 0.05) at baseline. Depression in ESAS-r had moderate correlations with PHQ-9 (r = 0.68) and HADS-Depression (r = 0.60). Anxiety in ESAS-r had moderate correlations with HADS-Anxiety (r = 0.65).

**FIGURE 4:**
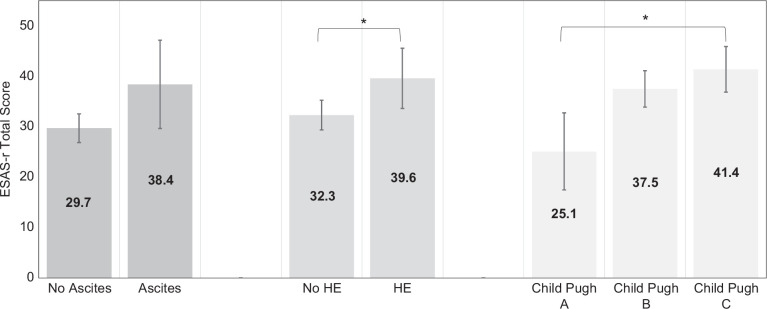
Known-groups validity of ESAS-r among 218 patients with decompensated cirrhosis. *p < 0.05. Abbreviation: ESAS-r, Edmonton Symptom Assessment System; HE, hepatic encephalopathy.

Known-groups validity. There were statistically significant differences in ESAS-r total scores for patients with versus without HE (39.6 vs. 32.3, *p* = 0.016) and with increasing severity of liver disease (Child-Pugh A: 25.1; B: 37.5; C: 41.4; *p* = 0.006), but not for patients with versus without ascites (38.4 vs. 29.7, *p* = 0.084) (Figure 4). There were clinically significant differences in ESAS-r total scores (based on MCID: 3–4 points) among all groups.

### Responsiveness

ESAS-r total scores significantly changed with clinically meaningful changes in PHQ-9 (MCID: 5 points) from baseline to week 12, with a mean difference of −10.3 (95% CI: −15.9, −4.7) for those with ≥ 5-point decrease in PHQ-9 and a mean difference of 14.5 (95% CI: 7.5, 21.5) in those with ≥ 5-point increase in PHQ-9. For patients without a clinically meaningful change in PHQ-9, the mean difference in ESAS-r total scores was not significant (−1.4 [95% CI: −4.7, 1.8]). Similar findings were found for the responsiveness of ESAS-r to clinically meaningful changes in HADS-Depression and HADS-Anxiety (MCID: 1.5 points) and SF-LDQOL (MCID: 8 points) as shown in Table 3.

**TABLE 3:** Mean differences in ESAS-r total score between baseline and week 12 by minimal clinically important differences in other PROMs using complete case analysis^a^

		ESAS-r total score (mean [SD])		
	n	Baseline	Week 12	Mean difference (95% CI)
PHQ-9 (n = 123)				
≤−5	22	49.2 (16.7)	38.4 (18.9)	**−10.3** (**−15.9, −4.7)**
−4 to 4	87	32.3 (19.3)	30.6 (19.8)	−1.4 (−4.7, 1.8)
≥5	14	34.7 (13.4)	51.1 (15.0)	**14.5** (**7.5, 21.5)**
HADS-Depression (n = 131)				
≤ −1.5	32	39.3 (22.7)	30.4 (21.0)	**−8.2** (**−14.3, −2.1)**
−1.4 to 1.4	59	32.1 (18.7)	32.5 (20.1)	0.4 (−3.9, 4.8)
≥1.5	40	37.6 (17.1)	41.9 (19.3)	**3.9** (**0.0, 7.7)**
HADS-Anxiety (n = 133)				
≤−1.5	40	41.5 (19.1)	32.1 (18.2)	**−8.9** (**−13.6, −4.2)**
−1.4 to 1.4	60	31.3 (20.3)	30.8 (20.5)	−0.5 (−4.9, 3.9)
≥1.5	33	36.9 (15.7)	44.8 (19.5)	**7.3** (**3.0, 11.5)**
SF-LDQOL (n = 123)				
≤−8	34	34.0 (19.0)	43.2 (22.4)	**8.6** (**2.8, 14.3)**
−7 to 7	43	34.6 (18.8)	34.8 (19.7)	0.2 (−3.2, 3.6)
≥8	46	40.2 (21.2)	27.3 (18.8)	**−12.3** (**−18.3, −6.2)**

Statistically significant values are in bold.

a In the 135 patients who were eligible for the week 12 assessment, 12 patients were missing PHQ-9; 4 patients for HADS-Depression; and 2 patients for HADS-Anxiety.

Abbreviations: ESAS-r, revised Edmonton Symptom Assessment System; HADS, Hospital Anxiety and Depression Scale; PHQ-9, Patient Health Questionnaire-9; PROMs, patient-reported outcome measures; SF-LDQOL, Short Form Liver Disease Quality of Life.

### Predictive validity

In univariable regression analysis, we found that change in symptom burden as measured by ESAS-r explained 24% of the variance in SF-LDQOL scores from baseline to week 12 (Table 4). After adjusting for demographic and clinical covariates, the change of ESAS-r was still significantly correlated with the change of SF-LDQOL (β = −0.37, *p* < 0.001; Table 4). The adjusted *R* ^2^ was 0.30. In the sensitivity analysis (Supplemental Table E2, http://links.lww.com/HC9/A797) in which the total score of SF-LDQOL was calculated without sexual functioning, the results were consistent with the main findings and did not change the interpretation.

**TABLE 4:** Predictors of change in SF-LDQOL by the change of ESAS-r from baseline to week 12 (N = 122)^a^

	Univariate regression analysis			Multivariable analysis		
Predictors	*β (SE)*	*R* ^*2*^	*p*	*β (SE)*	*R* ^*2*^	*p*
	—	—	—	—	0.30	—
Age at baseline	−0.01 (0.11)	0.00	0.922	0.05 (0.10)	—	0.632
MELD-Na score at baseline	−0.03 (0.20)	0.00	0.873	0.00 (0.18)	—	0.995
Ascites	−2.02 (3.60)	0.00	0.577	−3.97 (3.36)	—	0.239
HE	−1.59 (2.54)	0.00	0.533	−0.33 (2.34)	—	0.889
Alcohol-associated cirrhosis	0.99 (2.19)	0.00	0.653	2.69 (2.03)	—	0.188
Listed for transplant at enrollment	−4.64 (2.16)	0.04	**0.033** ^b^	−4.35 (2.08)	—	**0.038** ^b^
HCC at enrollment	1.44 (3.60)	0.00	0.691	5.08 (3.64)	—	0.165
CirCom Score	−0.60 (1.07)	0.00	0.574	−1.07 (1.12)	—	0.342
Change in ESAS-r score	−0.36 (0.06)	0.24	**<0.001** ^b^	−0.37 (0.06)	—	**<0.001** ^b^

Statistically significant values are in bold.

a In the 135 patients who were eligible for the week 12 assessment, 6 were missing the change score of ESAS-r; 6 were missing the change score of SF-LDQOL; and 1 was missing the baseline MELD-Na score.

b *p* < 0.05.

Abbreviations: CirCom, cirrhosis-specific comorbidity scoring system; ESAS-r, revised Edmonton Symptom Assessment System; MELD-Na, Model for End-Stage Liver Disease-Sodium Score; SF-LDQOL, Short Form Liver Disease Quality of Life.

## DISCUSSION

The aim of this study was to assess the psychometric properties of the ESAS-r in a longitudinal cohort of 218 adult patients with DC. Our patient cohort had high disease severity: 59% were Child-Pugh B, 32% were Child-Pugh C, the Median MELD-Na score of the cohort was 16, and 50% were listed for liver transplantation. Our patient cohort also had a high symptom burden, with over 50% reporting moderate-to-severe tiredness, drowsiness, poor well-being, and pain. In this study, we found that the ESAS-r is a reliable and valid measure that demonstrates robust internal consistency, structural validity, convergent validity, and known-groups validity in a heterogenous population with DC. Despite our patient cohort having high rates of moderate-to-severe symptom burden across multiple symptoms, we did not find any significant floor or ceiling effects in the ESAS-r total score. Importantly, we found that total symptom burden as measured by ESAS-r has strong predictive validity for the change in patients’ HRQOL longitudinally as measured by SF-LDQOL and was responsive to clinically meaningful changes in patient-reported depression, anxiety, and HRQOL. To our knowledge, this study is the first to perform a rigorous psychometric assessment of the ESAS-r in adult patients with DC, establishing it as a reliable, valid, and responsive symptom assessment tool that can be used as a quality measure in routine cirrhosis care or as an outcome measure in future clinical trials.

To our knowledge, our work is the first to demonstrate the relative contribution of symptom burden to HRQOL among patients with DC. We found that ESAS-r total scores were strongly correlated with HRQOL as measured by SF-LDQOL and additionally, changes in symptom burden accounted for 30% of the changes in patients’ HRQOL longitudinally. The relative contribution of symptom burden to HRQOL has been previously noted in other populations with chronic diseases, including chronic kidney disease and cancer.^8^,^29^,^30^ Notably, MELD-Na scores had a very weak correlation with SF-LDQOL scores for patients in this study. These results are consistent with a growing body of evidence suggesting biological disease severity might not correlate with patients’ HRQOL.^8^,^15^,^31^,^32^ Symptom burden and HRQOL are separate but intimately related constructs. Symptom burden refers to the intensity, frequency, and impact of physical and psychological symptoms experienced by an individual and is one important dimension of HRQOL, which also encompasses other dimensions such as functional, social, and cognitive well-being. In turn, symptom burden is proximal to, and an important determinant of, HRQOL. Furthermore, easy-to-use and simple-to-interpret PROMs assessing symptom burden such as ESAS-r can provide more immediately actionable information to clinicians caring for patients as compared to the dimensions reported in an HRQOL questionnaire. Overall, our findings suggest that multidimensional symptom management could significantly improve the HRQOL of patients with DC.

The potential utility of ESAS-r to improve comprehensive symptom screening and to facilitate longitudinal symptom monitoring in routine cirrhosis care is immense. The ESAS-r is a simple PROM with strong content validity and an easy-to-use numerical scale that patients and clinicians alike find easy to administer, interpret, and score.^7^ It has been cross-culturally adapted and validated and is currently available in over 20 languages, facilitating its administration in diverse populations. Recent data show that the ESAS-r takes less than 1–2 minutes to complete on average, even among patients with advanced cancer who have low health literacy.^33^ The brevity of the ESAS-r also allows for its ease of integration through electronic data capture and for electronic symptom monitoring even within large health networks, as has been implemented at a population level in Ontario for patients receiving cancer care.^34^,^35^ Cirrhosis care currently focuses greatly on managing liver disease complications, but there is no standardized tool to screen for and/or monitor physical and psychological symptom burden. In clinical practice, the ESAS-r can be easily used to rapidly identify not only patients with DC experiencing multiple distressing symptoms but also to tailor symptom interventions for individual patients. The ESAS-r can subsequently be used to monitor for symptom improvement after treatment.^7^ Future work should evaluate the feasibility of routine symptom monitoring using the ESAS-r in patients with DC.

The ESAS-r also shows great promise as a clinical endpoint in cirrhosis clinical trials. The ESAS-r has a well-established MCID for both individual symptom scores and its total score and its validation in the population with DC supports its use in symptom management intervention trials.^3^,^5^,^6^ Importantly, the ESAS-r can be used as a key outcome measure to capture the effect of interventions that have the potential to address multiple symptoms simultaneously, such as cognitive behavioral therapy, integrative medicine, and palliative care. Longitudinal symptom monitoring, particularly when associated with triggered alerts to clinicians, has been shown to improve symptoms and HRQOL, reduce acute care utilization, and even increase quality-adjusted survival among adults with cancer in multiple randomized controlled trials.^36^,^37^,^38^,^39^ Lastly, the ESAS-r has been shown to have predictive validity for outcomes such as acute care use in patients with head and neck cancers and more recently among patients with cirrhosis.^40^,^41^,^42^ Assessing the use of ESAS-r as a predictor of not just health care utilization in patients with DC but also outcomes such as transplant-free survival and overall survival warrant further investigation.

While this study did establish the reliability, validity, and responsiveness of the ESAS for adult outpatients with DC, it does have several limitations. First, patients were recruited from a single liver transplant center and were 90% White, limiting generalizability. However, our cohort comprised a socioeconomically diverse population with substantial heterogeneity in cirrhosis etiology and disease severity. Second, the ESAS-r is a 10-item questionnaire that may not capture all symptoms that patients with DC may experience such as pruritis or sexual dysfunction. However, the ESAS-r allows for clinicians to include a tenth symptom, such as muscle cramps as was done in this study, to allow for customization for individual patients. Third, we only collected MELD-Na scores at baseline and not at week 12 as this was not routinely available for most of the patients in our study. Future research is needed to validate the longitudinal relationship between the MELD-Na score and ESAS-r. Fourth, we did not conduct test-retest reliability within a short time window as the ESAS-r has already demonstrated high test-retest reliability (intraclass correlation coefficients ≥0.7) among other populations with serious illness such as in end-stage renal disease and cancer.^7^,^8^,^31^,^43^ Fifth, we did not formally screen for the presence of HE during week 12 assessments. Last, while we were able to establish the external responsiveness of ESAS-r total scores using the external anchors of clinically meaningful changes in PHQ-9, HADS, and SF-LDQOL, our study design did not include an intervention that allowed us to determine internal responsiveness.

## CONCLUSION

The ESAS-r is a reliable, valid, and responsive tool for longitudinally assessing symptom burden in patients with DC, which is predictive of changes in HRQOL. Future directions include the implementation of the ESAS-r in clinical practice and research as a key outcome measure in cirrhosis care and clinical trials.

## Supplementary Material

## References

[1] Peng JK Hepgul N Higginson IJ Gao W. Symptom prevalence and quality of life of patients with end-stage liver disease: A systematic review and meta-analysis. Palliat Med. 2019;33:24–36. 30345878 10.1177/0269216318807051 PMC6291907

[2] Hansen L Chang MF Hiatt S Dieckmann NF Lyons KS Lee CS. Symptom frequency anddistress underestimated in decompensated cirrhosis. Dig Dis Sci. 2022;67:4234–442. 34448980 10.1007/s10620-021-07216-7 PMC8882195

[3] Patel AA Tapper EB Kanwal F Woodrell CD Hansen L Lai JC. Targets and study design for symptom-focused trials aimed at patients with cirrhosis: An expert consensus. Hepatol Commun. 2023;7:e0135. 37267219 10.1097/HC9.0000000000000135 PMC10241502

[4] Rogal SS Hansen L Patel A Ufere NN Verma M Woodrell CD. AASLD Practice Guidance: Palliative care and symptom-based management in decompensated cirrhosis. Hepatology. 2022;76:819–853. 35103995 10.1002/hep.32378 PMC9942270

[5] Patel AA Woodrell C Ufere NN Hansen L Tandon P Verma M. Developing priorities for palliative care research in advanced liver disease: A multidisciplinary approach. Hepatol Commun. 2021;5:1469–1480. 34510839 10.1002/hep4.1743 PMC8435283

[6] Verma S Hingwala J Low JTS Patel AA Verma M Bremner S. Palliative clinical trials in advanced chronic liver disease: Challenges and opportunities. J Hepatol. 2023;79:1236–1253. 37419393 10.1016/j.jhep.2023.06.018

[7] Hui D Bruera E. The Edmonton Symptom Assessment System 25 years later: Past, present, and future developments. J Pain Symptom Manage. 2017;53:630–643. 28042071 10.1016/j.jpainsymman.2016.10.370 PMC5337174

[8] Davison SN Jhangri GS Johnson JA. Longitudinal validation of a modified Edmonton symptom assessment system (ESAS) in haemodialysis patients. Nephrol Dial Transplant. 2006;21:3189–3195. 16957010 10.1093/ndt/gfl380

[9] Mazzaferro V Regalia E Doci R Andreola S Pulvirenti A Bozzetti F. Liver transplantation for the treatment of small hepatocellular carcinomas in patients with cirrhosis. N Engl J Med. 1996;334:693–699. 8594428 10.1056/NEJM199603143341104

[10] Jepsen P Vilstrup H Lash TL. Development and validation of a comorbidity scoring system for patients with cirrhosis. Gastroenterology. 2014;146:147–156; quiz e15-6. 24055278 10.1053/j.gastro.2013.09.019

[11] Marchesini G Bianchi G Amodio P Salerno F Merli M Panella C. Factors associated with poor health-related quality of life of patients with cirrhosis. Gastroenterology. 2001;120:170–178. 11208726 10.1053/gast.2001.21193

[12] Chatrath H Liangpunsakul S Ghabril M Otte J Chalasani N Vuppalanchi R. Prevalence and morbidity associated with muscle cramps in patients with cirrhosis. Am J Med. 2012;125:1019–1025. 22835465 10.1016/j.amjmed.2012.03.012 PMC3932181

[13] Hui D Shamieh O Paiva CE Khamash O Perez-Cruz PE Kwon JH. Minimal clinically important difference in the physical, emotional, and total symptom distress scores of the Edmonton Symptom Assessment System. J Pain Symptom Manage. 2016;51:262–269. 26482223 10.1016/j.jpainsymman.2015.10.004 PMC4733575

[14] Gralnek IM Hays RD Kilbourne A Rosen HR Keeffe EB Artinian L. Development and evaluation of the Liver Disease Quality of Life instrument in persons with advanced, chronic liver disease--the LDQOL 1.0. Am J Gastroenterol. 2000;95:3552–3565. 11151892 10.1111/j.1572-0241.2000.03375.x

[15] Kanwal F Spiegel BMR Hays RD DURAZO F HAN SB SAAB S. Prospective validation of the short form Liver Disease Quality of Life instrument. Aliment Pharmacol Ther. 2008;28:1088–1101. 18671776 10.1111/j.1365-2036.2008.03817.x

[16] Kanwal F Gralnek IM Hays RD Zeringue A Durazo F Han SB. Health-related quality of life predicts mortality in patients with advanced chronic liver disease. Clin Gastroenterol Hepatol. 2009;7:793–799. 19306949 10.1016/j.cgh.2009.03.013

[17] Zigmond AS Snaith RP. The hospital anxiety and depression scale. Acta Psychiatr Scand. 1983;67:361–370. 6880820 10.1111/j.1600-0447.1983.tb09716.x

[18] Kroenke K Spitzer RL Williams JBW. The PHQ-9: validity of a brief depression severity measure. J Gen Intern Med. 2001;16:606–613. 11556941 10.1046/j.1525-1497.2001.016009606.x PMC1495268

[19] Terwee CB Bot SDM de Boer MR van der Windt DAWM Knol DL Dekker J. Quality criteria were proposed for measurement properties of health status questionnaires. J Clin Epidemiol. 2007;60:34–42. 17161752 10.1016/j.jclinepi.2006.03.012

[20] McHorney CA Tarlov AR. Individual-patient monitoring in clinical practice: Are available health status surveys adequate? Qual Life Res. 1995;4:293–307. 7550178 10.1007/BF01593882

[21] Prinsen CAC Mokkink LB Bouter LM Alonso J Patrick DL de Vet HCW. COSMIN guideline for systematic reviews of patient-reported outcome measures. Qual Life Res. 2018;27:1147–1157. 29435801 10.1007/s11136-018-1798-3 PMC5891568

[22] Wang J Wang X. Structural Equation Modeling: Applications Using Mplus. New York, USA: John Wiley & Sons Ltd; 2019.

[23] Muthen L Muthen B. Mplus User’s Guide. Los Angeles, USA: Muthen & Muthen. 2017.

[24] Schober P Boer C Schwarte LA. Correlation coefficients: Appropriate use and interpretation. Anesth Analg. 2018;126:1763–1768. 29481436 10.1213/ANE.0000000000002864

[25] Husted JA Cook RJ Farewell VT Gladman DD. Methods for assessing responsiveness: A critical review and recommendations. J Clin Epidemiol. 2000;53:459–468. 10812317 10.1016/s0895-4356(99)00206-1

[26] Löwe B Unützer J Callahan CM Perkins AJ Kroenke K. Monitoring depression treatment outcomes with the patient health questionnaire-9. Med Care. 2004;42:1194–1201. 15550799 10.1097/00005650-200412000-00006

[27] Puhan MA Frey M Büchi S Schünemann HJ. The minimal important difference of the hospital anxiety and depression scale in patients with chronic obstructive pulmonary disease. Health Qual Life Outcomes. 2008;6:46. 18597689 10.1186/1477-7525-6-46 PMC2459149

[28] Younossi ZM Golabi P Henry L. A comprehensive review of patient-reported outcomes in patients with chronic liver diseases. J Clin Gastroenterol. 2019;53:331–341. 30702486 10.1097/MCG.0000000000001179

[29] Astrup GL Rustøen T Hofsø K Gran JM Bjordal K. Symptom burden and patient characteristics: Association with quality of life in patients with head and neck cancer undergoing radiotherapy. Head Neck. 2017;39:2114–2126. 28766791 10.1002/hed.24875

[30] de Ligt KM Heins M Verloop J Ezendam NPM Smorenburg CH Korevaar JC. The impact of health symptoms on health-related quality of life in early-stage breast cancer survivors. Breast Cancer Res Treat. 2019;178:703–711. 31512091 10.1007/s10549-019-05433-3 PMC6817812

[31] Davison SN Jhangri GS Johnson JA. Cross-sectional validity of a modified Edmonton symptom assessment system in dialysis patients: A simple assessment of symptom burden. Kidney Int. 2006;69:1621–1625. 16672923 10.1038/sj.ki.5000184

[32] Saab S Ibrahim AB Shpaner A Younossi ZM Lee C Durazo F. MELD fails to measure quality of life in liver transplant candidates. Liver Transpl. 2005;11:218–223. 15666392 10.1002/lt.20345

[33] Wong A Tayjasanant S Rodriguez-Nunez A Park M Liu D Zapata KP. Edmonton Symptom Assessment Scale time duration of self-completion versus assisted completion in patients with advanced cancer: A randomized comparison. Oncologist. 2021;26:165–171. 33252169 10.1002/onco.13619 PMC7873322

[34] Barbera L Lee F Sutradhar R. Use of patient-reported outcomes in regional cancer centres over time: A retrospective study. CMAJ Open. 2019;7:E101–E108. 10.9778/cmajo.20180074 PMC6380903 30782773

[35] Pereira J Green E Molloy S Dudgeon D Howell D Krzyzanowska MK. Population-based standardized symptom screening: Cancer Care Ontario’s Edmonton Symptom Assessment System and performance status initiatives. J Oncol Pract. 2014;10:212–214. 24756143 10.1200/JOP.2014.001390

[36] Basch E Deal AM Kris MG Scher HI Hudis CA Sabbatini P. Symptom monitoring with patient-reported outcomes during routine cancer treatment: A randomized controlled trial. J Clin Oncol. 2016;34:557–565. 26644527 10.1200/JCO.2015.63.0830 PMC4872028

[37] Basch E Deal AM Dueck AC Scher HI Kris MG Hudis C. Overall survival results of a trial assessing patient-reported outcomes for symptom monitoring during routine cancer treatment. JAMA. 2017;318:197–198. 28586821 10.1001/jama.2017.7156 PMC5817466

[38] Basch E Schrag D Henson S Jansen J Ginos B Stover AM. Effect of Electronic symptom monitoring on patient-reported outcomes among patients with metastatic cancer: A randomized clinical trial. JAMA. 2022;327:2413–2422. 35661856 10.1001/jama.2022.9265 PMC9168923

[39] Strasser F Blum D von Moos R Cathomas R Ribi K Aebi S. The effect of real-time electronic monitoring of patient-reported symptoms and clinical syndromes in outpatient workflow of medical oncologists: E-MOSAIC, a multicenter cluster-randomized phase III study (SAKK 95/06). Ann Oncol. 2016;27:324–332. 26646758 10.1093/annonc/mdv576

[40] Deng LX Kent DS O'Riordan DL Pantilat SZ Lai JC Bischoff KE. Symptom burden is associated with increased emergency department utilization among patients with cirrhosis. J Palliat Med. 2022;25:213–218. 34348042 10.1089/jpm.2021.0219 PMC8861938

[41] Noel CW Sutradhar R Zhao H Delibasic V Forner D Irish JC. Patient reported symptom burden as a predictor of emergency department use and unplanned hospitalization in head and neck cancer: A longitudinal population-based study. J Clin Oncol. 2021;39:675–684. 33405964 10.1200/JCO.20.01845

[42] Noel CW Forner D Chepeha DB Baran E Chan KKW Parmar A. The Edmonton Symptom Assessment System: A narrative review of a standardized symptom assessment tool in head and neck oncology. Oral Oncol. 2021;123:105595. 34775181 10.1016/j.oraloncology.2021.105595

[43] Mori M Morita T Yokomichi N Nitto A Takahashi N Miyamoto S. Validation of the Edmonton Symptom Assessment System: Ascites modification. J Pain Symptom Manage. 2018;55:1557–1563. 29581035 10.1016/j.jpainsymman.2018.03.016

