# Supplementary material for: The Edmonton Symptom Assessment System is a valid, reliable, and responsive tool to assess symptom burden in decompensated cirrhosis
Source: Hepatol Commun. 2024 Mar 18;8(4):e0385. doi: 10.1097/HC9.0000000000000385 (PMC10948137; doi:10.1097/HC9.0000000000000385)
Supplement: Supplementary file 1 [file hc9-8-e0385-s001.docx]

**APPENDICES**

**Appendix A: Demographics**

Please check the appropriate box or boxes.

1. Gender

- Man
- Woman
- Other

1. Ethnicity

- Hispanic or Latino
- Not Hispanic or Latino

1. Race (please check all that apply)

- American Indian or Alaskan native
- Asian
- African American or Black
- Native Hawaiian or other Pacific Islander
- White
- Other (please specify) _____________

1. Religion

- Catholic Christian
- Other Christian (such as Protestant, Orthodox, etc.)
- Jewish
- Muslim
- Atheist
- None
- Other (please specify)_______________

1. Current relationship status

- Married or living with someone as if married
- Non-cohabiting relationship
- Single, never married
- Divorced/Separated
- Loss of long term partner/ Widowed

1. Please indicate your highest or current education level

- 11^th^ grade or less
- High school graduate or GED
- 2 years of college/AA degree/Technical school training
- College graduate (BA or BS)
- Masters degree
- Doctorate/Medical degree/Law degree

1. What is your annual combined household income?

- Less than $25,000
- $25,000 – 50,000
- $50,000 -100,000
- $100,000 – 150,000
- Greater than $150,000

1. Please indicate who you live with (you may check more than one box)

- By myself
- Partner/Spouse
- Roommate/Friend
- Children under 18
- Children over 18
- Group home/assisted living/nursing home
- Parent
- Other (please specify) _____________

1. Current employment status

(please check all that apply):

- Employed (full-time or part-time)
- Caring for home or family (not currently working and not looking for paid work)
- Unemployed and looking for work
- Unable to work due to illness or disability
- Retired
- Student
- Other (please specify) _____________

**Appendix B: Short Form of Liver Disease Quality of Life Instrument (SF-LDQOL)**


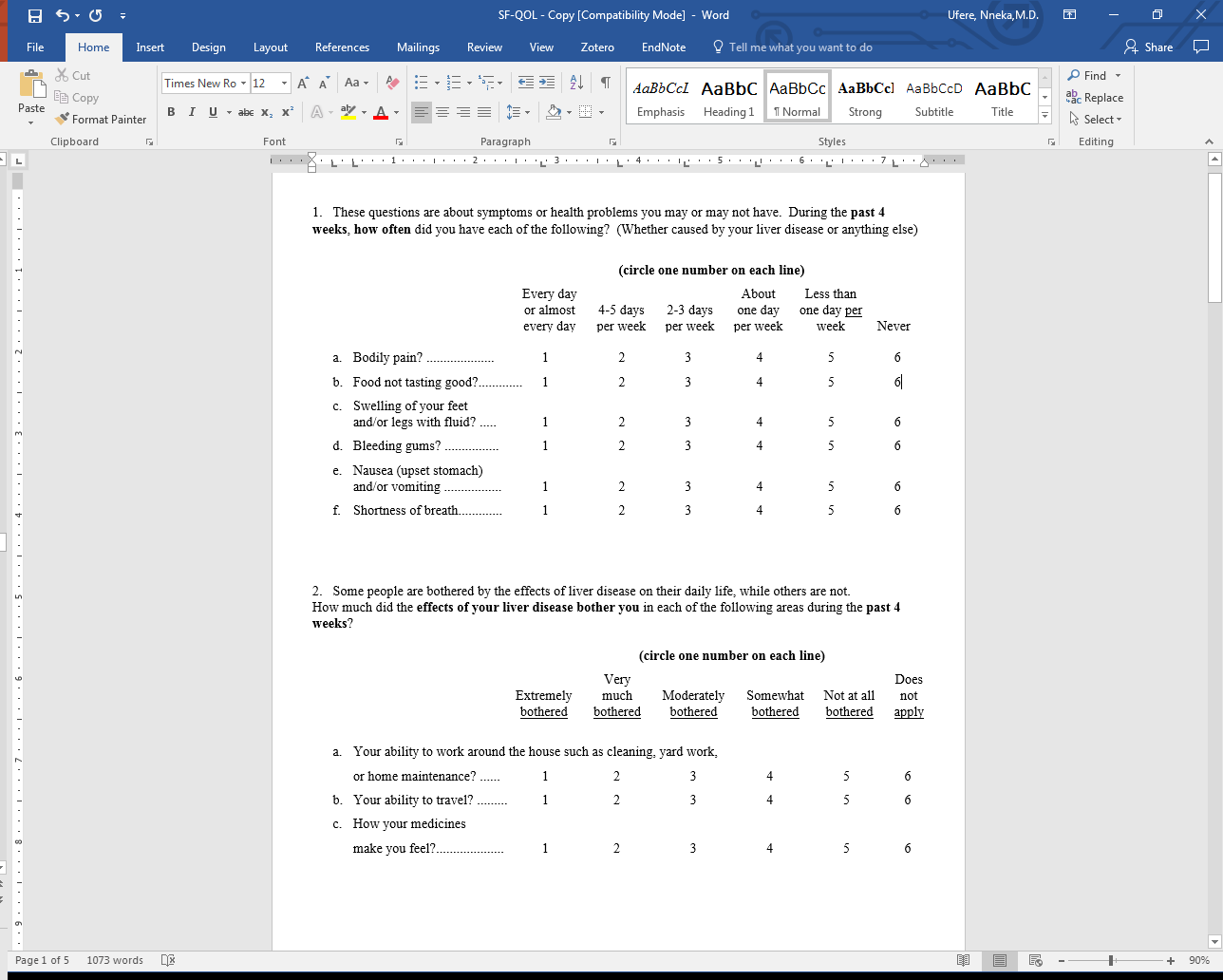


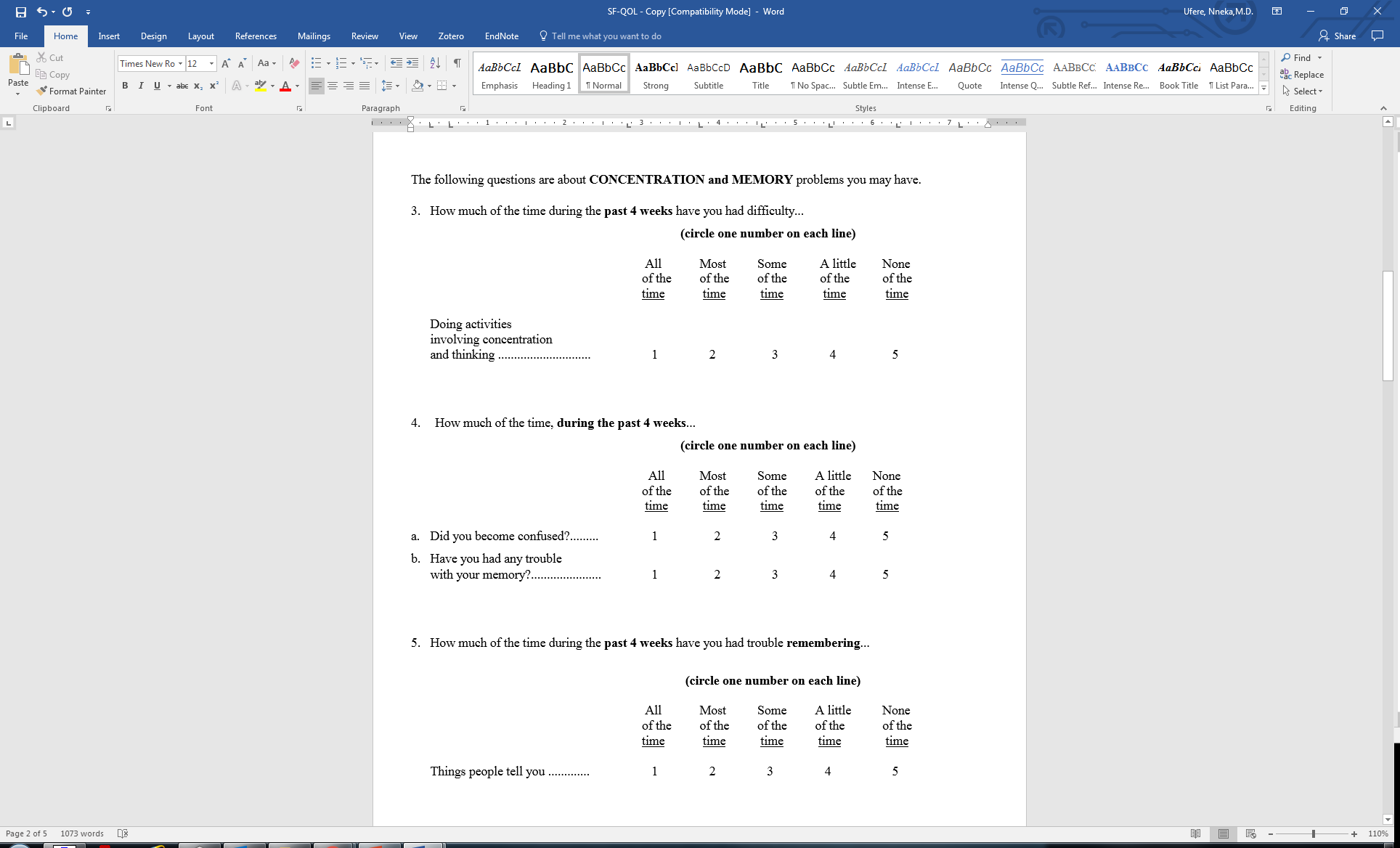


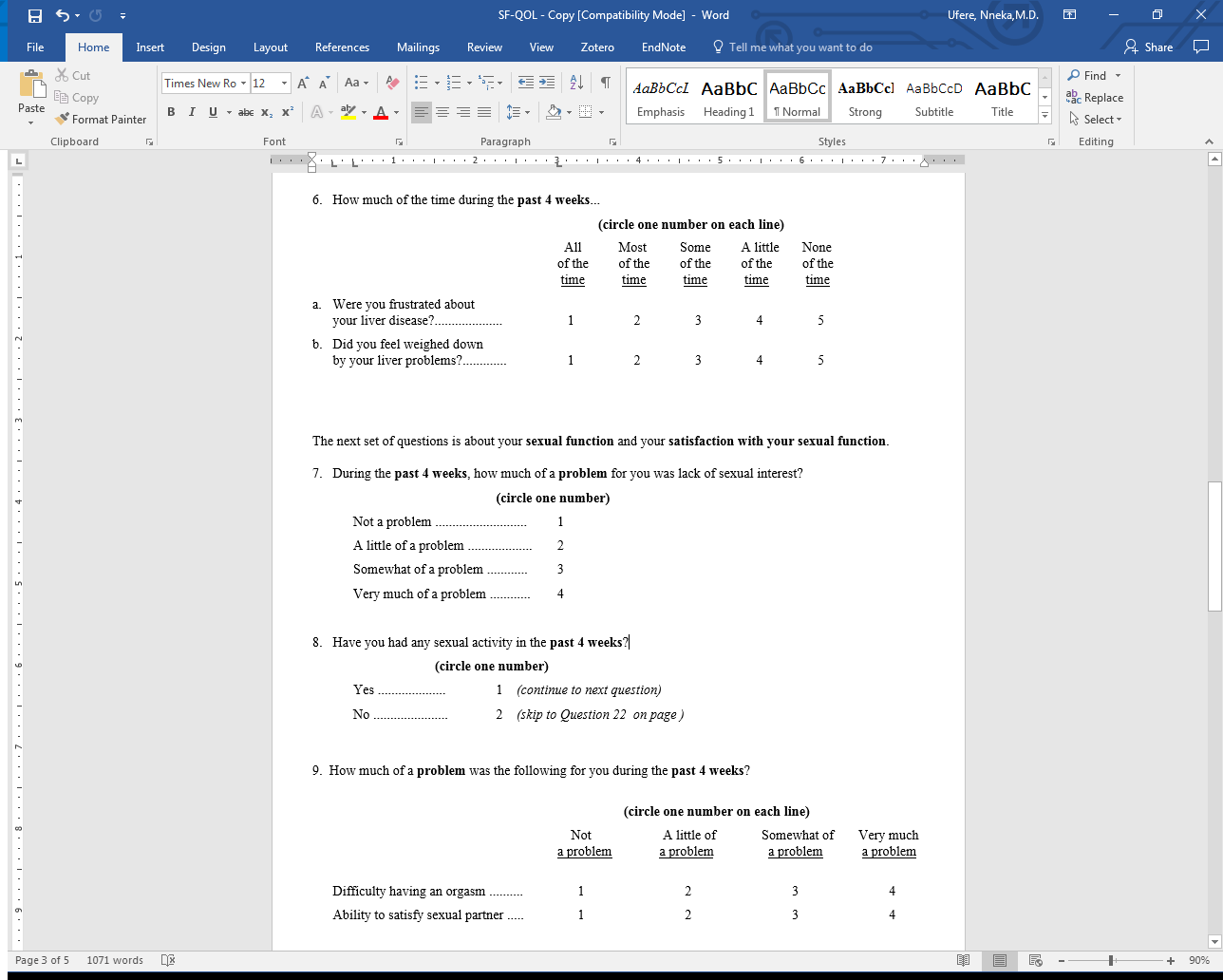


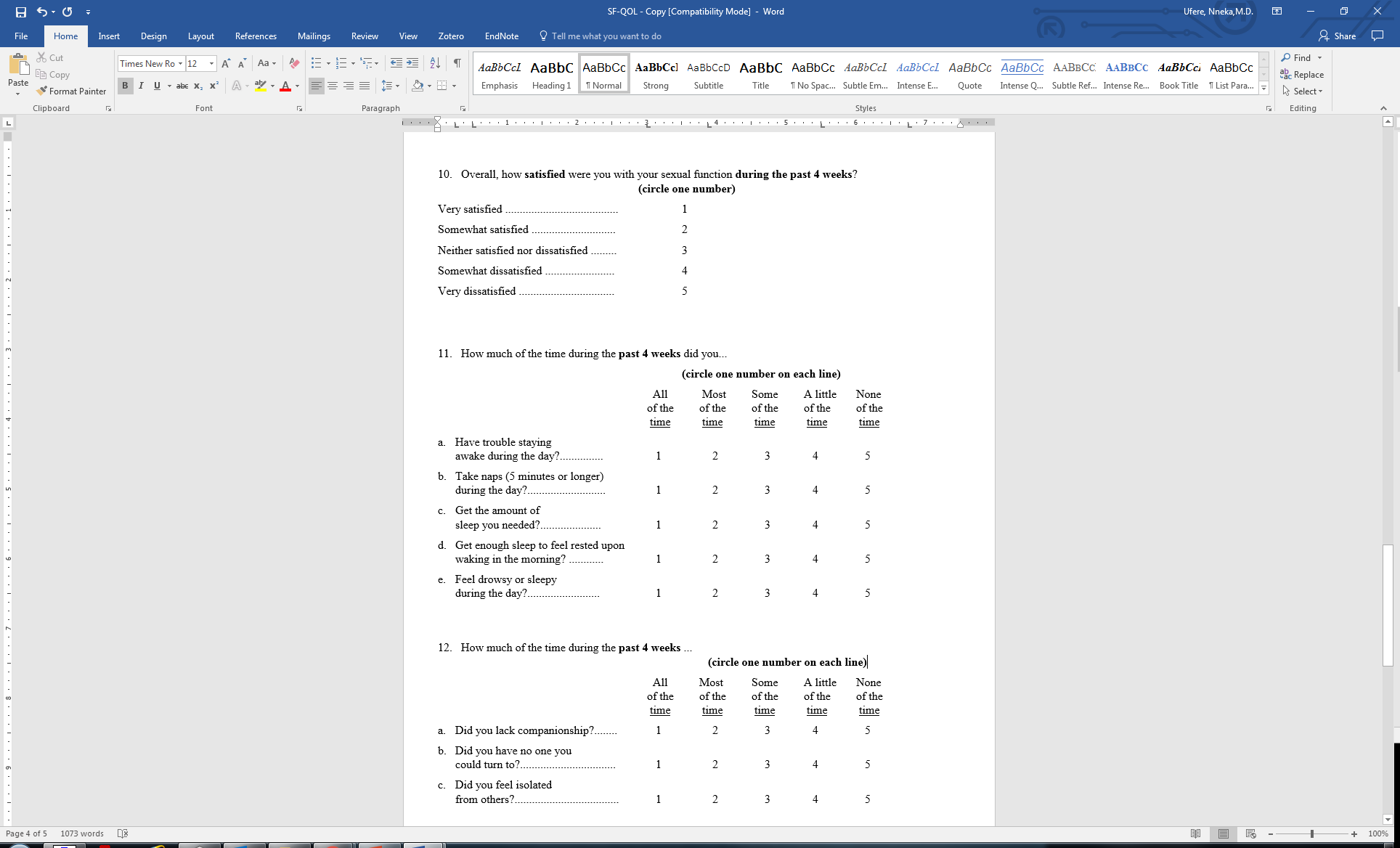


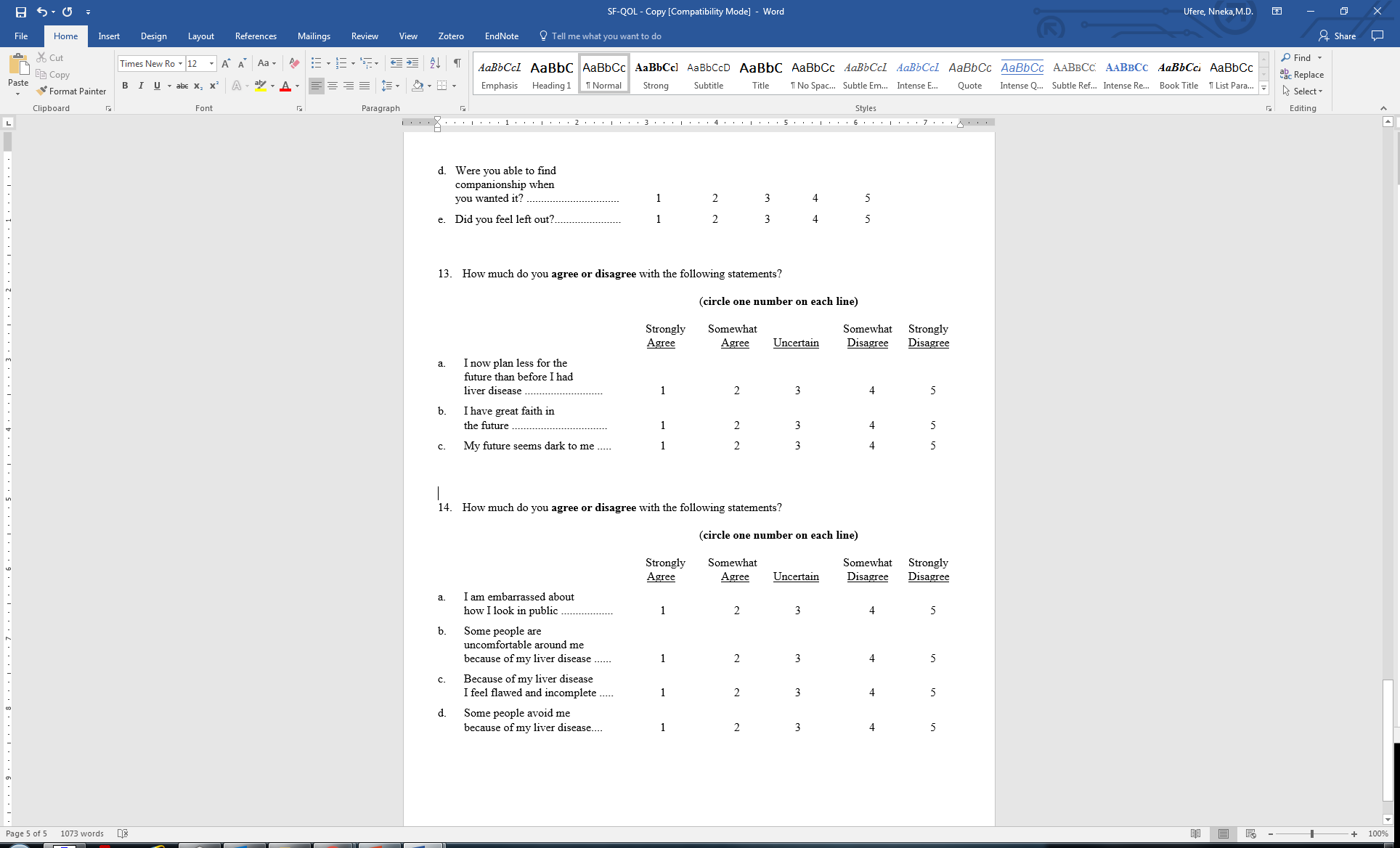


**Appendix C: Revised Edmonton Symptom Assessment Scale (ESAS-r)**


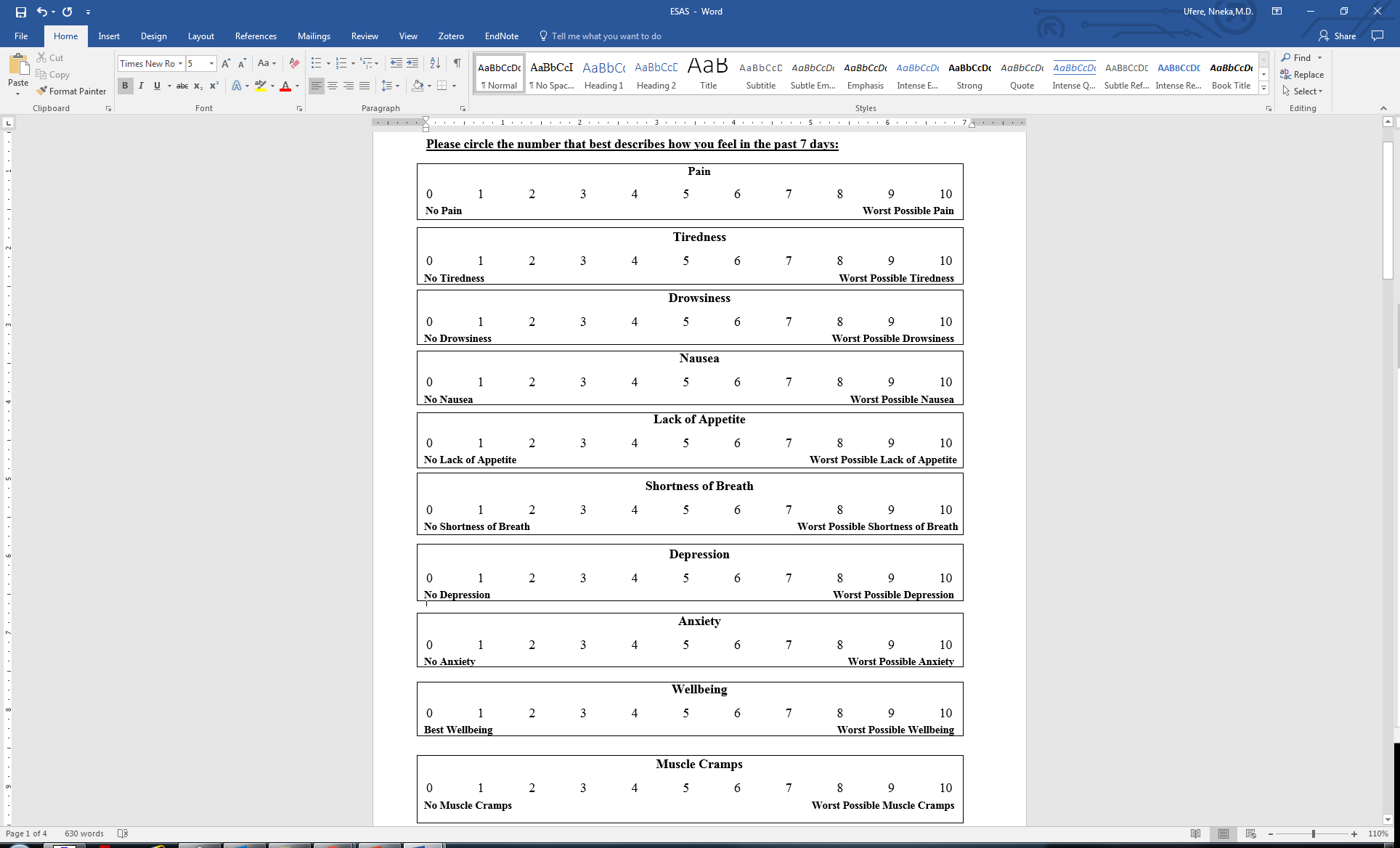


**Appendix E: PHQ-9**

Over the **last** **two weeks** have you been bothered by the following problems?

(Please answer by circling **one** answer for each problem.)

|  | **Not at all** | **Several Days** | **More than half the days** | **Nearly everyday** |
| --- | --- | --- | --- | --- |
| Little interest or pleasure in doing things. | **1** | **2** | **3** | **4** |
| Feeling down, depressed, or hopeless. | **1** | **2** | **3** | **4** |
| Trouble falling or staying asleep, or sleeping too much. | **1** | **2** | **3** | **4** |
| Feeling tired or having little energy. | **1** | **2** | **3** | **4** |
| Poor appetite or overeating. | **1** | **2** | **3** | **4** |
| Feeling bad about yourself, or that you are a failure, or have let your family down. | **1** | **2** | **3** | **4** |
| Trouble concentrating on things such as reading. | **1** | **2** | **3** | **4** |
| Moving or speaking so slowly that other people could have noticed. Or the opposite—being so fidgety or restless that you have been moving around a lot more than usual. | **1** | **2** | **3** | **4** |
| Thoughts that you would be better off dead. | **1** | **2** | **3** | **4** |
