# Supplementary material for: The Edmonton Symptom Assessment System is a valid, reliable, and responsive tool to assess symptom burden in decompensated cirrhosis
Source: Hepatol Commun. 2024 Mar 18;8(4):e0385. doi: 10.1097/HC9.0000000000000385 (PMC10948137; doi:10.1097/HC9.0000000000000385)
Supplement: Supplementary file 2 [file hc9-8-e0385-s002.docx]

**Supplemental Table E1.** Frequencies of moderate-to-severe symptoms^†^ at baseline (N=218)*

| **Symptoms** | **N (%, 95%CI)** |
| --- | --- |
| Pain | 109 (50.0 [43.4, 56.6]) |
| Tiredness | 167 (76.6 [70.5, 81.8]) |
| Drowsiness | 149 (68.4 [61.9, 74.2]) |
| Nausea (n=216) | 73 (33.8 [27.8, 40.3]) |
| Lack of appetite (n=217) | 82 (37.8 [31.6, 44.4]) |
| Shortness of breath (n=217) | 67 (30.9 [25.1, 37.3]) |
| Depression | 76 (34.9 [28.8, 41.4]) |
| Anxiety (n=217) | 96 (44.2 [37.8, 50.9]) |
| Poor wellbeing (n=216) | 120 (55.6 [48.9, 62.0]) |
| Muscle cramps (n=217) | 103 (47.5 [40.9, 54.1]) |

†: Moderate-to-severe symptom burden was defined as an individual item score equal to or larger than 4.

*: unless specified. Otherwise, the sample size is 218.

**Supplemental Table E2.** Predictors of change in SF-LDQOL^†^ by the change of ESAS-r (N=122)‡

| **Predictors** | **Univariate regression analysis** | | | **Multivariable analysis** | | |
| --- | --- | --- | --- | --- | --- | --- |
|  | ***β (SE)*** | ***R^2^*** | ***p*** | ***β (SE)*** | ***R^2^*** | ***p*** |
|  |  |  |  |  | 0.31 |  |
| Age at baseline | 0.01 (0.10) | 0.00 | 0.899 | 0.07 (0.09) |  | 0.461 |
| MELD-Na score at baseline | -0.01 (0.19) | 0.00 | 0.972 | 0.02 (0.17) |  | 0.906 |
| Ascites | -2.29 (3.42) | 0.00 | 0.504 | -3.65 (3.13) |  | 0.247 |
| Hepatic encephalopathy | -1.53 (2.41) | 0.00 | 0.527 | -0.21 (2.19) |  | 0.923 |
| Alcohol-related cirrhosis | -0.53 (2.08) | 0.00 | 0.800 | 0.95 (1.90) |  | 0.619 |
| Listed for transplant at enrollment | -4.72 (2.04) | 0.04 | **0.022*** | -4.54 (1.94) |  | **0.021*** |
| HCC at enrollment | 0.66 (3.42) | 0.00 | 0.846 | 3.83 (3.39) |  | 0.262 |
| CirCom score | -0.58 (1.02) | 0.00 | 0.569 | -0.82 (1.05) |  | 0.435 |
| Change in ESAS-r score | -0.36 (0.06) | 0.26 | **<0.001*** | -0.36 (0.06) |  | **<0.001*** |

*: p<0.05

†: Sexual functioning was excluded from the calculation of SF-LDQOL total score.

‡: Out of the 135 patients who were eligible for the week 12 assessment, 6 were missing the change score of ESAS-r; 6 were missing the change score of SF-LDQOL; and 1 was missing the baseline MELD-Na score.

SF-LDQOL, Short-Form Liver Disease Quality of Life questionnaire; ESAS-r, revised Edmonton Symptom Assessment System; MELD-Na, Model for End-Stage Liver Disease-Sodium Score; HCC, hepatocellular carcinoma; CirCom, cirrhosis-specific comorbidity scoring system
